# Analysis of Candidate miRNAs' Expression in Pancreatic Cancer

**DOI:** 10.1002/cam4.70400

**Published:** 2024-11-08

**Authors:** Rabeah Al‐Temaimi, Bicher Abdulkarim, Ali Al‐Ali, Bency John, Mrinmay Kumar Mallik, Kusum Kapila

**Affiliations:** ^1^ Human Genetics Unit, Department of Pathology, College of Medicine Kuwait University Jabriya Kuwait; ^2^ Undergraduate Medical Program, College of Medicine Kuwait University Jabriya Kuwait; ^3^ Department of Medicine, College of Medicine Kuwait University Jabriya Kuwait; ^4^ Department of Gastroenterology and Hepatology Amiri Hospital Kuwait City Kuwait; ^5^ Department of Pathology, College of Medicine Kuwait University Jabriya Kuwait; ^6^ Department of Laboratory Medicine Mubarak Al Kabeer Hospital Jabriya Kuwait

**Keywords:** KRAS, MicroRNA, NFKBIA, pancreatic cancer

## Abstract

**Background:**

Pancreatic cancer (PC) is one of the most aggressive types of cancer. Despite advances in molecular diagnostics, PC diagnosis relies on imaging technologies and morphological assessment of fine needle aspirates (FNAs). MicroRNA (miRNA) involvement in PC pathogenesis and potential diagnostics application have been suggested, albeit current supporting evidence is lacking. Here, we investigated the association of selected miRNAs with PC incidence and clinical characteristics.

**Methods:**

Fold expression of miR‐216a‐3p, ‐217‐5p, ‐221‐3p, ‐222‐3p, and miR‐196a‐5p was assessed in 73 PC FNA cell‐block sections and 6 healthy pancreas tissues using Taqman advanced miRNA assays. Potential miRNA targets were ascertained using immunocytochemistry.

**Results:**

miR‐196a‐5p was upregulated in PC compared to healthy pancreatic tissue (*β* = −0.05, 95% CI: −0.065 – (−0.035); *p* < 0.001). miR‐221‐3p and miR‐222‐3p fold expression were strongly correlated (*r* = 0.897, *p* < 0.001), whereas miR‐196a‐5p fold expression correlated with that of miR‐221‐3p (*r* = 0.688, *p* < 0.001) and miR‐222‐3p (*r* = 0.489, *p* < 0.001). Moreover, miR‐196a‐5p fold expression positively correlated with tumor stage (*r* = 0.32, *p* = 0.034). miR‐217‐5p fold expression inversely correlated with KRAS expression (*r* = ‐0.69, *p* = 0.0027).

**Conclusion:**

Our study shows miR‐196a‐5p has reasonable specificity to PC and thus may have diagnostic and prognostic potential in PC as proposed in the literature. Moreover, KRAS and NFKBIA may be potential targets for miR‐217‐5p and miR‐196a‐5p, respectively. Thus, these miRNAs may be involved in tumor progression and may have valuable applications in novel therapeutics or treatment monitoring.

## Graphical abstract

miR‐196a‐5p upregulation in pancreatic cancer is associated with cancer progression. miR‐217‐5p is downregulated in pancreatic cancer allowing for KRAS overexpression.

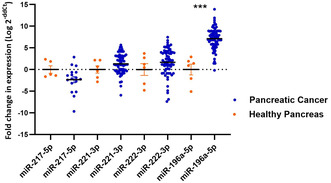

## 1 Introduction

MicroRNAs (miRNAs) are small (~22 nucleotides) non‐coding ribonucleic acid chains that serve as epigenetic silencing molecules of mRNA expression. The advantages of miRNAs as biomarkers for disease are concurrent with their physiological role. Such advantages include their structural stability and degradation resistance, rapid and transient expression in response to various physiological stimuli, and their high specificity to their targets and environmental conditions. Altered miRNA expression and regulation have been investigated in various cancers in the past decade, with few miRNAs used in clinical testing, specifically in personalized medicine [1, 2]. Nevertheless, miRNAs have provided insights into cancer pathogenesis and progression mechanisms that have greatly enhanced the diagnosis, prognosis, and monitoring of disease progression and response to treatment [3, 4, 5]. Among all cancers, pancreatic cancer is the most molecularly heterogeneous cancer, with a limited understanding of its molecular etiology and progression, primarily due to its diagnosis at the late stages of the disease.

Pancreatic cancer is one of the most aggressive cancers and accounts for 4.5% of all cancer deaths worldwide [6]. Pancreatic tumors can be classified into benign and malignant tumors. Malignant pancreatic cancers are categorized into two main categories: exocrine and neuroendocrine pancreatic cancers. Exocrine pancreatic cancers include pancreatic adenocarcinoma, adenosquamous carcinoma, squamous cell carcinoma, and colloid carcinoma. Pancreatic neuroendocrine tumors (PNET) are very rare and makeup < 5% of all pancreatic cancer diagnoses, whereas pancreatic adenocarcinoma or pancreatic ductal adenocarcinoma (PDAC) comprises 90% of all pancreatic cancer diagnoses with only 10% of PDACs diagnosed as inherited PDAC and 90% being sporadic cancers [7]. The global 5‐year survival rate for pancreatic cancer patients is currently 5.5%, a rate that has not changed markedly in the past decade despite advances in cancer therapies and surgical interventions. Pancreatic cancer's high mortality rate can be attributed to its extreme molecular heterogeneity, the difficulty of diagnosing it early, and the absence of standardized international guidelines in evaluating pancreatic masses when detected by imaging beyond morphological assessment by experienced cytopathologists. Despite advances in molecular diagnostic technologies, diagnosing pancreatic cancer still relies on imaging technologies, such as computed tomography and magnetic resonance imaging, and morphological assessment of fine needle aspirates (FNA). Both methods, however, are sought in active disease patients (advanced cancer stages) and have variable specificity and accuracy in pancreatic cancer. MiRNA applications in pancreatic cancer diagnosis have been suggested; however, current evidence is lacking in supporting their specificity to pancreatic cancer. Numerous exhaustive literature reviews have been conducted to identify miRNAs of potential clinical application from clinical samples and in vitro studies of pancreatic cancer (Table 1) [3, 8].

**TABLE 1:** A summary list of differentially expressed microRNAs in pancreatic cancer compared to normal pancreatic tissues [8].

MiRNA biomarkers	Level in pancreatic tissue	Level in pancreatic cancer
miRNA‐20a, ‐29c, ‐96, ‐141, ‐216^a^, ^b^, ‐217^a^, ^b^, ‐375^a^	High	Low
miRNA‐10a, ‐10b, ‐204, ‐372, ‐93^a^, ‐133a^a^, ‐203^a^, ‐205^a^, ‐210^a^, ‐224^a^, ‐27a, ‐221^a^, ^b^, ‐222^a^, ^b^, ‐15b, ‐95, ‐186, ‐190, ‐200b, ‐146a^b^, ‐143, ‐145, ‐150, ‐100, ‐125b‐1, ‐212, ‐301, ‐424, ‐21^b^, ‐155, ‐205	Low	High
miRNA‐196a^a^, ^b^	Absent	High

^a^ Corroborating evidence from pancreatic cancer cell lines.

^b^ Replicated in at least one study.

Among these miRNAs, miR‐216, ‐217, ‐221, ‐222, and miR‐196a have been shown to have altered expression in pancreatic cancer patients' samples and experimental cell lines in at least two separate reports and were prioritized for our study [9, 10, 11]. For some of these miRNAs, functional evidence supporting their involvement in pancreatic cancer pathogenesis exists from functional studies of their potential targets in pancreatic cancer cell lines and animal models. Mainly, miR‐196a has been shown to target nuclear factor kappa‐B‐inhibitor alpha (*NFKBIA*) in pancreatic cancer cell lines [12]. Whereas miR‐216 and ‐217 are involved in pathways that regulate the expression of Kirsten rat sarcoma viral oncogene homolog (*KRAS*) [13, 14]. Our objective was to verify that reported PC miRNAs are altered in a well‐characterized understudied population sample and provide candidate target evidence to establish their potential role in the pathogenesis of PC.

## 2 Methods

### 2.1 Pancreatic Cancer Samples Collection

This study is a retrospective case‐cohort study in which archival clinical samples used were allocated and collected for research purposes that adhered to the World Medical Association Declaration of Helsinki‐Ethical Principles for Medical Research Involving Human Subjects and was granted the approval and waiver of patient consent by the Joint Committee for The Protection of Human Subjects at Kuwait's Ministry of Health (ERB number 3217). Eighty‐four archival formalin‐fixed paraffin‐embedded (FFPE) FNA cell blocks collected during 2016–2022 at Mubarak Al‐Kabeer Hospital were assessed for suitability for downstream analyses. Inclusion criteria of pancreatic cancer samples were based on the availability of demographic and clinical information and assessment of hematoxylin and eosin‐stained cell block sections. Inclusion criteria included having a clear pancreatic cancer type diagnosis, availability of demographic information, ascertained location of FNA sample (head, tail, or body of the pancreas), sufficient tumor cell material, and identified cell structures. Exclusion criteria included insufficient tumor cell material, absence of tumor cells, and undefined cellular structures due to FFPE artifacts. Tumor stage and grade were collected following the completion of the study experimental analyses to avoid any bias in the analysis. In total, 73 pancreatic cancer cases were selected, and six healthy pancreas samples were used as controls for miRNA expression analyses. Two commercial control pancreas total RNA samples (1 male, 1 female) and two male commercial healthy pancreas tissue slides (Zyagen, CA, USA; BioChain Institute Inc. CA, USA), in addition to two locally sourced healthy pancreas tissue blocks from the pancreas head (female) and the tail (male).

### 2.2 MiRNA Extraction

Six 10 μm thick sections were cut from each FNA cell block and mounted on slides. Focal tumor infiltrates were ascertained for each specimen using a hematoxylin and eosin stained guide section and were macro dissected using a sterile 21 gauge needle. Macro‐dissected sections were collected into a sterile 1.5 mL microcentrifuge tube containing 320 μL of xylene, followed by mixing for 10 s and brief centrifugation to pellet partially deparaffinized cells. Pellets were incubated in xylene at 56°C for 3 min, then allowed to cool at room temperature before miRNA extraction using Qiagen's miRNeasy FFPE kit (Qiagen, MD, USA) according to kit standard protocol. In brief, cells were lysed in 240 μL of PKD buffer by vortexing for 15 s. This was followed by centrifugation at 10,000 rpm for 1 min at room temperature. Ten microliters of proteinase K were added and mixed gently into the suspension by pipetting. The lysate was incubated at 56°C for 15 min followed by an incubation at 80°C for an additional 15 min. All incubations included interrupted mixing by vortexing every 5 min. Following incubation, the lower clear phase was transferred to a fresh 2 mL microcentrifuge tube and chilled on ice for 3 min, and then centrifuged at 13,500 rpm for 15 min. The supernatant was transferred without disturbing the debris pellet into a new microcentrifuge tube. DNase booster buffer was added in a volume equal to a tenth of the supernatant volume followed by 10 μL of DNase I solution. The suspension was mixed by gentle inversions and briefly spun down to collect. The reaction was incubated at room temperature for 15 min. Five hundred microliters of RBC buffer was mixed into the suspension followed by 1750 μL of absolute ethanol and mixed by pipetting. The mixture was then loaded into RNeasy MinElute spin columns and centrifuged at 11,000 rpm for 15 s. Columns were washed with 500 μL RPE buffer twice with centrifugation at 11,000 rpm for 15 s and 2 min, respectively. After the final wash columns were dried by spinning open capped at maximum speed for 5 min. Bound miRNAs were eluted into a sample ID labeled microcentrifuge using 25 μL of elution buffer followed by centrifugation at full speed for 1 min. Extracts were assessed for quantity using a spectrophotometer.

### 2.3 MiRNA Reverse Transcription and Amplification

For the detection and quantification of mature miRNAs in samples TaqMan Advanced miRNA cDNA Synthesis Kit (ThermoFisher Scientific, MA, USA) was used according to manufacturer protocols. The multi‐step procedure involves poly‐A tailing, adaptor ligation, universal reverse transcription (RT), and a 14‐cycle amplification using 5 μL of RT product to allow detection of low transcript miRNA within the cycling conditions (miR‐Amp). In brief, the poly‐A tailing reaction contained 1X Poly(A) buffer, 1 mM dNTPs, 0.3 Unit/μL of poly(A) enzyme, and 2 μL of pancreatic cancer FNA miRNA extract and healthy control miRNA. The polyadenylation reaction was incubated at 37°C for 45 min and terminated at 65°C for 10 min. Reactions were cooled down on ice and the adaptor ligation reaction mix was added (1X DNA ligase buffer, 15%/μL polyethylene glycol (PEG), 1X ligation adaptor, and 1 Unit/μL RNA ligase). The adaptor ligation reaction was incubated at 16°C for 60 min. The universal reverse transcription (RT) reaction proceeded immediately by adding 1X RT buffer, 4 mM dNTPs, 1X RT enzyme mix, and 1X universal RT primer. The RT reaction was incubated at 42°C for 15 min and terminated by incubation at 85°C for 5 min. Lastly, the 14‐cycle amplification step was performed using 5 μL of universal RT product in a reaction containing 1X miR‐Amp master mix and 1X miR‐Amp primer mix for a final volume of 50 μL. The reaction was incubated at 95°C for 5 min, followed by 14 cycles of denaturation at 95°C for 3 s and annealing at 60°C for 30 s. The reaction was stopped by incubation at 99°C for 10 min. All incubations were performed in thermal cycler GeneAmp polymerase chain reaction (PCR) system 9700 (Applied Biosystems, CA, USA).

### 2.4 MiRNA Fold Expression Analysis

Semi‐quantitative or comparative expression Real‐Time PCR was performed on a 1:10 dilution of cDNA templates using TaqMan's human advanced miRNA assays (ThermoFisher Scientific, MA, USA) for each of the five chosen miRNAs (miR‐216a‐3p assay ID: 478770_mir, miR‐217‐5p assay ID: 478773_mir, miR‐221‐3p assay ID: 477981_mir, miR‐222‐3p assay ID: 477982_mir, and miR‐196a‐3p assay ID: 478745_mir) and snoRNA U91 (Assay ID: Hs03298712_s1) as endogenous reference control [15]. In brief, each reaction contained 5 μL of TaqMan Fast Advanced Master Mix (ThermoFisher Scientific, MA, USA), 0.5 μL of TaqMan advanced miRNA assay, 2 μL of RNase free water, and 2.5 μL of cDNA template. All reactions were run in triplicates for both control pancreas and pancreatic cancer samples. Reactions were run on ABI 7500 Fast Real‐time PCR system (Applied Biosystems, CA, USA), and the delta–delta Ct method (2^−∆∆Ct^) was used to determine selected miRNA levels in pancreatic cancer in comparison to healthy pancreas levels.

### 2.5 Immunocytochemistry

Pancreatic cancer FNA cell blocks were sectioned into 4‐5 μm thin sections and stained with hematoxylin and eosin to confirm tumor cell presence and absence of any fixation artifacts. Each section was washed in xylene three times for 3‐min incubation intervals, followed by 3 washes in a descending Alcohol series. Slides were rinsed with deionized water and antigen retrieval was performed by boiling in 0.01 M citrate buffer pH 6.0. After cooling at room temperature, slides were washed in deionized water and immersed in Tris‐Buffered Saline (TBS) for 5 min. Blocking was done using a serum‐free protein block (Agilent, CA, USA) for 30 min at room temperature followed by overnight incubation with primary antibodies at 4°C. PathPlus polyclonal Rabbit anti‐Human KRAS antibody (LS‐A10587, LSBio, MA, USA) and polyclonal Rabbit anti‐Human NFKBIA (LS‐C331335, LSBio, MA, USA) were used at 1:100 dilution. Slides were washed in three changes of TBS pH 7.6 the next day and incubated with the secondary antibody (Dako REAL EnVision Detection System, Agilent, CA, USA) for 1 h at room temperature. The slides were washed in TBS for 5 min and blocked with 3% H_2_O_2_ for 10 min and washed in water. Diaminobenzidine (DAB) (Agilent Technologies, Glostrup, Denmark) was added and incubated for 2–5 min, then washed with deionized water. Slides were counterstained with Hematoxylin for 30 s and washed in tap water, dehydrated in an ascending alcohol series, and cleared in 3 changes of xylene and mounted in DPX. All viewing and image capture were performed on Axio Imager.A1 (Carl Zeiss, Gottingen, Germany).

### 2.6 Statistical Analysis

MiRNA expression analysis was performed using the relative gene expression or comparative Ct method. Raw 2^−∆∆Ct^ fold expression results were checked for normality and lognormality using the Shapiro–Wilk test. Results were Log transformed to achieve normal distribution and direction of change. Student *t*‐test was performed on Log‐transformed data, in addition to linear regression analysis. Clinicopathological variables were analyzed using linear and logistic regression analyses. Pearson correlation was performed to determine the association of tumor stage and grade with miRNA fold expression. Staining intensity was scored according to the following negative (0: no staining), weakly positive (1+: pale orange granular), moderately positive (2+: orange), and strongly positive (3+: dark brownish orange). All statistical analyses were conducted using SPSS v.26 (IBM, NY, USA).

## 3 Results

### 3.1 miRNA Expression in Pancreatic Cancer

Seventy‐three pancreatic cancer FNA cell blocks were deemed suitable for miRNA extraction where the tumor cellularity exceeded normal cells. Table 2 shows the included samples' demographics and clinical characteristics.

**TABLE 2:** Demographics and clinical characteristics of pancreatic cancer samples.

Criteria	Pancreatic cancer samples (*n* = 73)
Sex assigned at birth [*n* (%)]	
Male	52 (71.2)
Female	21 (28.8)
Age in years [mean ± standard deviation]	63.7 ± 11.1
Nationality [*n* (%)]	
Kuwaiti	36 (49.3)
Non‐Kuwaiti	37 (50.7)
Cytological diagnosis [*n* (%)]	
Ductal adenocarcinoma	71 (97.3)
Adenocarcinoma foamy cell variant	2 (2.2)
Tumor location [*n* (%)]	
Head and uncinate	53 (72.6)
Body and tail	20 (27.4)
Tumor Stage [*n* (%)]	
2	2 (2.7)
3	7 (9.6)
4	35 (48)
Not available	29 (39.7)
Tumor Grade [*n* (%)]	
1	26 (35.6)
2	31 (42.5)
3	5 (6.8)
Not available	11 (15.1)
Necrosis [*n* (%)]	
0	16 (21.9)
1	15 (20.5)
2	18 (24.7)
3	13 (17.8)
Not available	11 (15.1)
KRAS mutations (*n* = 48)	
G12	7 (14.6)
G13	3 (6.25)
Q61	9 (18.75)
Negative	29 (60.4)

**FIGURE 1:**
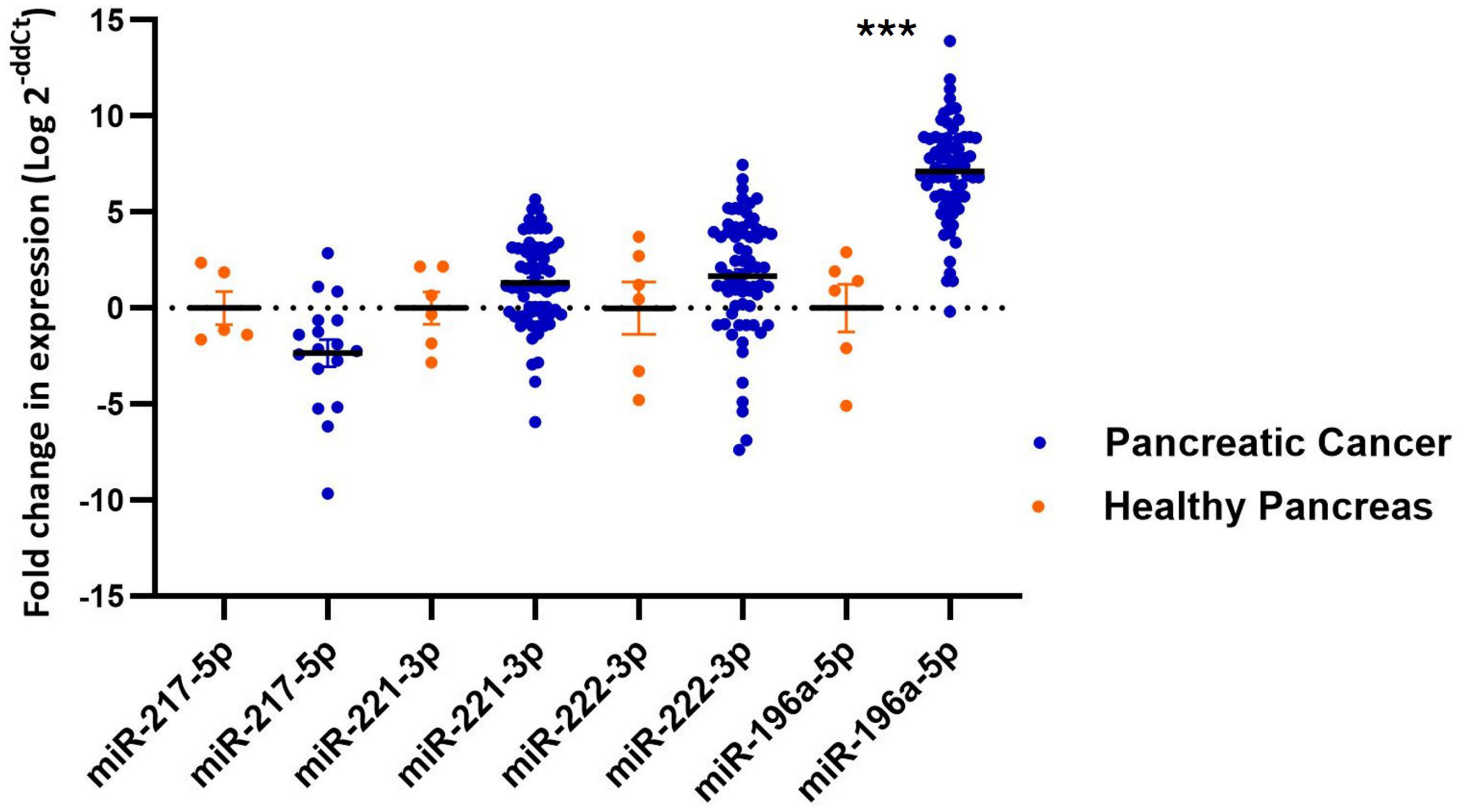
A scatterplot of Log2 transformed 2^−ddCt^ values of miRNAs' fold expression change in pancreatic cancer compared to Healthy pancreas tissues. The horizontal bar indicates the mean, error bars indicate ± standard error of mean (SEM), and (*) denotes statistically significant association from a student *t*‐test analysis.

MiRNA expression fold changes in pancreatic adenocarcinoma FNA compared to healthy pancreas tissues are shown in Table 3 (Figure 1). MiR‐216a‐3p was completely undetected in PDAC samples, whereas in healthy control pancreas samples, it was detected in only one sample. MiR‐217‐5p was detected in 17 PDAC samples, with a trend of reduced expression in most samples except for three specimens, which showed a positive increase compared to normal pancreas levels. MiR‐221‐3p was neither statistically differentially expressed between PDAC and healthy control samples, nor was miR‐222‐3p. MiR‐196a‐5p was the only miRNA with a statistically significant expression fold change compared to healthy pancreatic tissue expression (*β* = 0.05, 95% CI: 0.035–0.065; *p* < 0.001). Comparing miRNA fold expression changes to each other, miR‐221‐3p and miR‐222‐3p fold expressions were strongly correlated (*r* = 0.897, *p* < 0.001). Similarly, miR‐196a‐5p fold expression strongly correlated with miR‐221‐3p (*r* = 0.688, *p* < 0.001) and moderately with miR‐222‐3p (*r* = 0.489, *p* < 0.001) fold expressions.

**TABLE 3:** Log2 transformed means of miRNAs' fold expression change (2^−ddCt^) ± standard error of mean (SEM) in healthy pancreatic tissues (HC) compared to pancreatic cancer tissues (PC). The *p*‐values are a result of student *t*‐test statistical analyses.

miRNA	Number expressed		Mean fold expression (±SEM)		*p*
	HC	PC	HC	PC	
hsa‐miR‐216a‐3p	1	0	0	Undetected	—
hsa‐miR‐217‐5p	5	17	0.0 (±0.86)	−2.35 (±0.723)	0.115
hsa‐miR‐221‐3p	6	73	1.83^e‐6^ (±0.84)	1.315 (±0.275)	0.178
hsa‐miR‐222‐3p	6	73	−1.33^e‐6^ (±1.37)	1.658 (±0.36)	0.21
hsa‐miR‐196a‐5p	6	73	−1.33^e‐6^ (±1.23)	7.11 (±0.29)	< 0.0001

None of the demographics or pathomorphological characteristics assessed correlated with miRNA fold expressions except for the weak positive correlation between miR‐196a‐5p fold expression and tumor stage (*r* = 0.32, *p* = 0.034; Table 4).

**TABLE 4:** Expression fold change of analyzed miRNAs with pancreatic adenocarcinoma stage.

MiRNA	Stage 2 (*n* = 2)	Stage 3 (*n* = 7)	Stage 4 (*n* = 35)
hsa‐miR‐217‐5p	Undetected	Undetected	−1.37
hsa‐miR‐221‐3p	0.29	1.0	1.69
hsa‐miR‐222‐3p	2.08	0.47	1.8
hsa‐miR‐196a‐5p	5.42	6.04	7.75

### 3.2 MiRNA Target Assessment in Pancreatic Cancer

**FIGURE 2:**
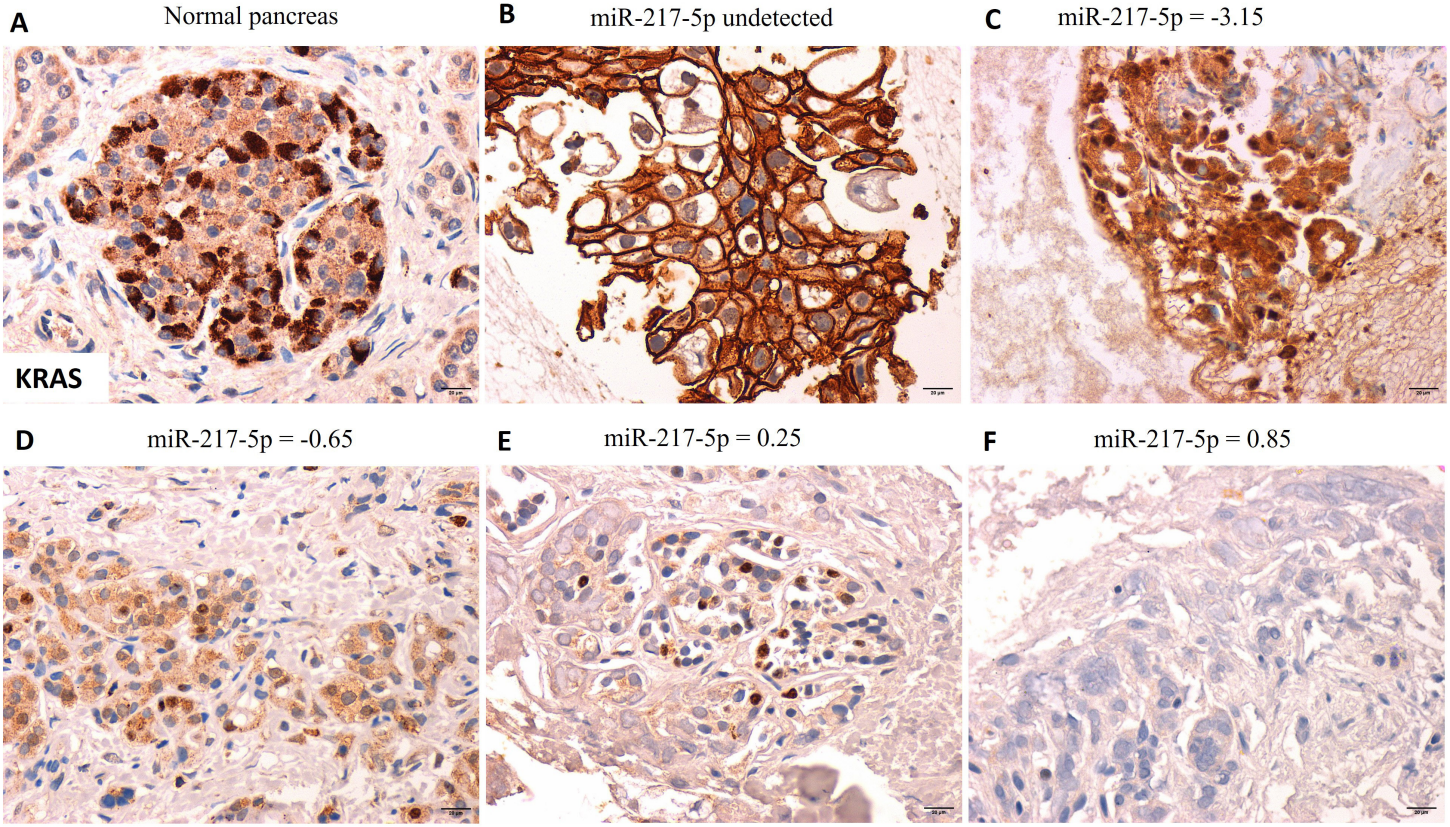
KRAS expression in normal pancreas (A) in comparison to pancreatic ductal carcinoma fine needle aspirate cell block sections (B–F) and their respective miR‐217‐5p fold expression. All images were captured at 400× magnification, and scale bars denote 20 μm.

MiR‐217‐5p was differently expressed in 17 pancreatic cancer FNA samples assessed. KRAS expression is regulated by miR‐217‐5p [14]. We assessed KRAS expression in all 17 samples with sufficient tumor cell infiltrates (Table 5). Figure 1 shows examples of KRAS protein expression with miR‐217‐5p fold expression. All PDAC specimens included were stage 4, except for one stage 3 PDAC. A negative correlation between miR‐217‐5p fold change and KRAS protein expression was statistically significant (*r* = −0.694, *p* = 0.0027). KRAS expression in normal pancreas tissue (Figure 2A) was most intensely in the islets of Langerhans with cytoplasmic localization and at lower intensities in ductal and acinar cells. In PDAC samples, KRAS expression and localization differed. The miR‐217‐5p undetected sample was an undifferentiated PDAC with mostly membranous KRAS expression (Figure 2B), whereas in low miR‐217‐5p PDACs, a cytoplasmic KRAS expression was noted with sporadic nuclear staining (Figure 2C,D). Meanwhile, in miR‐217‐5p high expressing PDACs, KRAS staining was either nuclear or undetected (Figure 2E,F). Only two PDAC specimens were positive for KRAS mutations and had detectable miR‐217‐5p expression. One specimen with KRAS G12V mutation had 2.7 fold decrease in miR‐217‐5p expression and high expression of KRAS in the nucleus and cytoplasm. Whereas the second specimen had G13R KRAS mutation and a miR‐217‐5p 0.85 increase in fold expression, in which KRAS protein expression was negative (Figure 2F).

**TABLE 5:** KRAS protein expression and localization in PDAC with the fold change in expression of miR‐217‐5p.

miR‐217‐5p fold expression	KRAS staining intensity	Location
−9.65	2	Nuclear/Cytoplasm
−6.15	2	Cytoplasm
−5.25	1	Cytoplasm
−3.15	2	Cytoplasm
−2.75	3	Cytoplasm
−2.4	3	Nuclear/Cytoplasm
−2.25	1	Nuclear/Cytoplasm
−2.15	1	Cytoplasm
−1.9	3	Nuclear/Cytoplasm
−1.38	1	Cytoplasm
−1.25	2	Cytoplasm
−0.65	1	Cytoplasm
−0.65	1	Cytoplasm
0	3	Membranous/Cytoplasm
0.25	0	—
0.85	0	—
1.1	0	—

**FIGURE 3:**
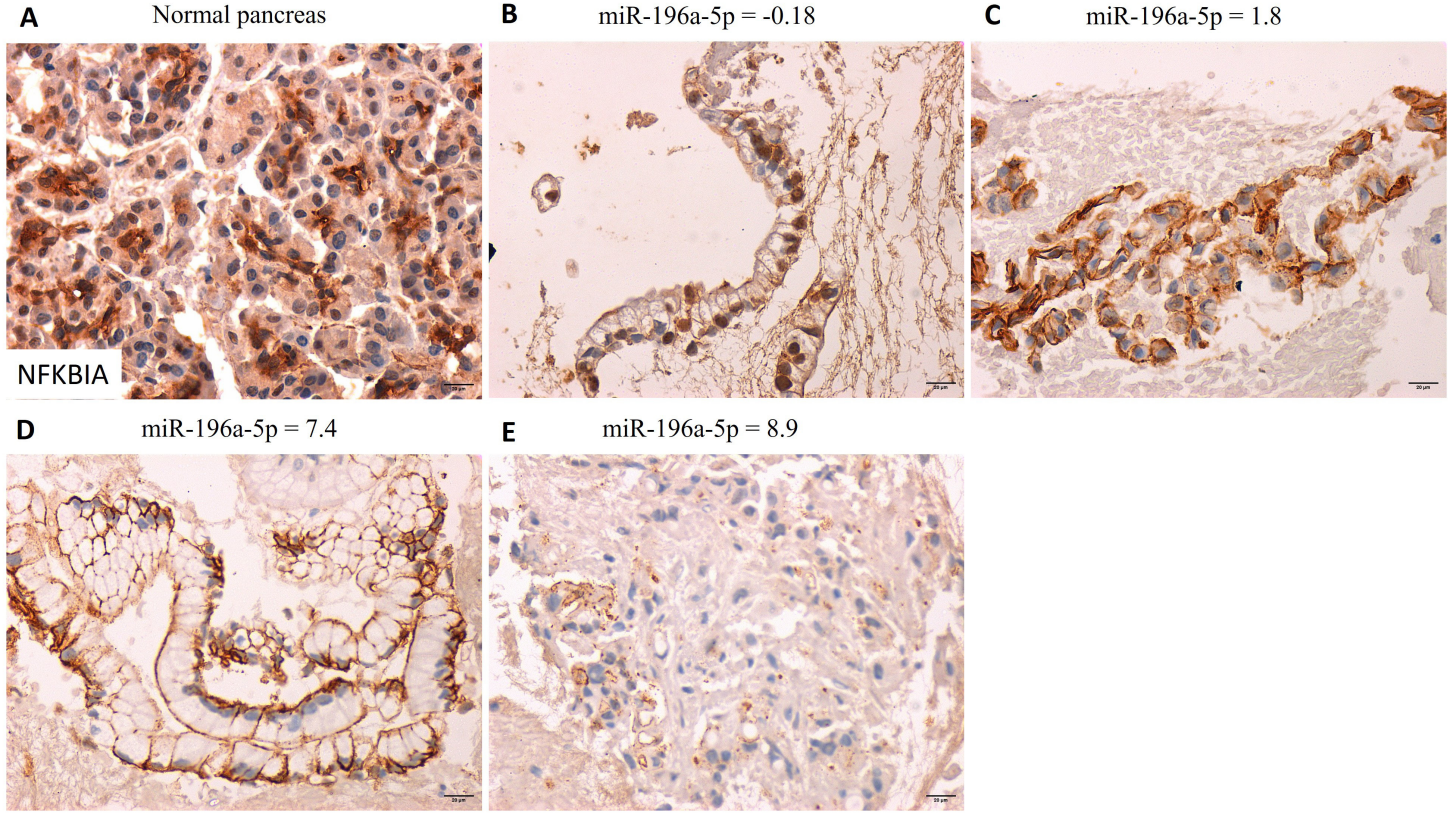
NFKBIA expression in normal pancreas (A) in comparison to pancreatic ductal carcinoma fine needle aspirate cell block sections (B–E) and their respective miR‐196a‐5p fold expression. All images were captured at 400× magnification, and scale bars denote 20 μm.

NFKBIA was suggested as a target for miR‐196a‐5p, therefore we analyzed its expression in PDAC samples (Figure 3). In the normal pancreas, NFKBIA expression showed nuclear/cytoplasmic localization (Figure 3A). In miR‐196a‐5p low expressing well‐differentiated PDAC, the localization of NFKBIA was mostly nuclear and membranous (Figure 3B). In miR‐196a‐5p 1.8fold expressing PDAC, NFKBIA nuclear and cytoplasmic localization was lost (Figure 3C). However, in high miR‐196a‐5p expressing well‐differentiated PDAC NFKBIA expression was membranous (Figure 3D), whereas in high miR‐196a‐5p expressing undifferentiated PDAC, NFKBIA showed mild punctate membranous staining (Figure 3E).

## 4 Discussion

There are no current biomarkers for the early detection of pancreatic cancer that are of clinical use due primarily to its low incidence and ambiguous early presentation. However, several molecular biomarkers have been suggested to monitor disease progression and/or response to tailored treatments. MiRNA biomarkers have been suggested as potentially valuable disease detection and progression biomarkers that are not fully explored yet [16, 17]. Many potential miRNAs have been reported for their association with several PDAC characteristics, however, these potential miRNAs are results of mostly underpowered or in vitro studies or are rarely replicated in other studies to substantiate their validity. Here, we selected miRNAs that have corroborating studies of their involvement in pancreatic cancer pathogenesis or progression and show their association and potential targets in PDAC.

MiR‐216a was completely undetected or of low expression beyond our assay detection limit in our PDAC cohort, similar to reported findings [9, 11, 18]. MiR‐216a has been suggested to have a tumor suppressor function inhibiting the expression of many proteins of different subclasses that are involved in pancreatic cancer pathogenesis [19]. However, unlike previous reports, we only detected miR‐216a‐3p in one healthy pancreas sample, specifically in the pancreas head sample, suggesting that miR‐216a absence may not be specific to PDAC. On the other hand, miR‐217 had detectable low fold expression in 23.3% of our PDAC samples, similar to previous reports [18, 20]. MiR‐217 is also a tumor suppressor miRNA, inhibiting the expression of proteins involved in PDAC epithelial‐mesenchymal transition, invasion, and metastasis [21, 22]. The most prominent potential miR‐217 mRNA target shown in vitro is the oncogene *KRAS* [14, 23]. We have shown some potential evidence of KRAS protein expression being influenced by miR‐217 fold expression, which may suggest that KRAS is a target for miR‐217 epigenetic silencing in PDAC clinical samples. This finding implies that using miR‐217 in targeted therapies for KRAS+ PDAC should be investigated [24].

MiR‐221 and ‐222 have highly similar sequences and have been reported to have oncogenic functions in many cancer types [25]. We found their expression to correlate with one another. However, their upregulation was not consistent across all PDACs, nor was it statistically significant compared to normal pancreas tissues. It has been suggested that the overexpression of these two miRNAs is associated with hyperplastic, high‐grade tumors for which we found no correlation [26]. Based on our results, the value of these miRNAs for PDAC prognosis, when considered alone, is of insufficient sensitivity or specificity for clinical use. Still, the benefits of silencing them in PDAC remain to be investigated [27]. The only miRNA that significantly discriminated between healthy pancreas and PDAC in our study was miR‐196a, and reports suggest its specificity to pancreatic cancer [9, 10]. Moreover, its expression correlated with the tumor stage and the expression of miR‐221 and ‐222. MiR‐196a was described to have an altered expression in sporadic and hereditary PDAC [28, 29], and its inclusion in a PDAC early detection biomarker panel has proven effective [30, 31]. A potential target of miR‐196a specific to PDAC is NFKBIA based on an in vitro study [12]. It was found that silencing NFKBIA by miR‐196a increases proliferation and migration. Our assessment of NFKBIA showed strong cytoplasmic and mild nuclear expression in normal pancreas, but its localization in PDAC differed. NFKBIA was moderately localized in the nucleus and plasma membrane in low miR‐196a expressing PDAC. However, in high miR‐196a expressing well‐differentiated PDAC, NFKBIA localization was membranous, and its intensity was moderate. The highest miR‐196a expression showed NFKBIA of mild intensity localized in a punctate pattern to the plasma membrane. These findings indicate that in PDAC, NFKBIA is retained in the nucleus and the plasma membrane in PDAC with a low miR‐196a expression. The retention of NFKBIA primarily in the plasma membrane in PDAC signifies the loss of its ability to perform its functions, which require shuttling between the cytoplasm and the nucleus to inhibit NF‐kappa‐B/REL complexes. Translocation of NF‐kappa‐B/REL complexes to the nucleus enables these complexes to activate the transcription of mitogenic and anti‐apoptotic proteins, thus evading pro‐inflammatory signals, immune surveillance, and promoting cancer cell proliferation [32].

Our study showed that miR‐196a is overexpressed in PDAC. However, its suggested use in PDAC prognosis could not be ascertained due to the lack of survival data for our study's cohort, which is one of the limitations of our study. Another limitation is the underrepresentation of early‐stage PDAC, which the rare incidence of early detection of PDAC can justify. An advantage of our study is the concurrent examination of miRNA fold expression and their suggested target proteins in patient PDAC FNA samples. In conclusion, we have corroborated evidence that some of these miRNAs are altered in PDAC and elaborated on their potential target expression effects. Further investigations into their candidacy as therapeutic targets or therapeutic tools (Antagomir) in PDAC are warranted.

## Author Contributions

**Rabeah Al‐Temaimi:** conceptualization (lead), data curation (lead), formal analysis (lead), funding acquisition (lead), investigation (lead), methodology (lead), project administration (lead), resources (lead), supervision (lead), writing – original draft (lead), writing – review and editing (lead). **Bicher Abdulkarim:** data curation (equal), investigation (equal). **Ali Al‐Ali:** data curation (equal), resources (equal), writing – review and editing (supporting). **Bency John:** investigation (equal), methodology (supporting). **Mrinmay Kumar Mallik:** data curation (equal), writing – review and editing (supporting). **Kusum Kapila:** data curation (supporting), formal analysis (supporting), investigation (supporting), writing – review and editing (supporting).

## Ethics Statement

Archival clinical specimens used in this study were granted a waiver of patient consent by the Joint Committee for The Protection of Human Subjects at Kuwait's Ministry of Health (ERB number 3217).

## Conflicts of Interest

The authors declare no conflicts of interest.

## Data Availability

The data underlying this article will be shared on reasonable request to the corresponding author.
